# Supplementary figures and images for: Soft disorder modulates the assembly path of protein complexes
Source: PLoS Comput Biol. 2022 Nov 17;18(11):e1010713. doi: 10.1371/journal.pcbi.1010713 (PMC9714922; doi:10.1371/journal.pcbi.1010713)

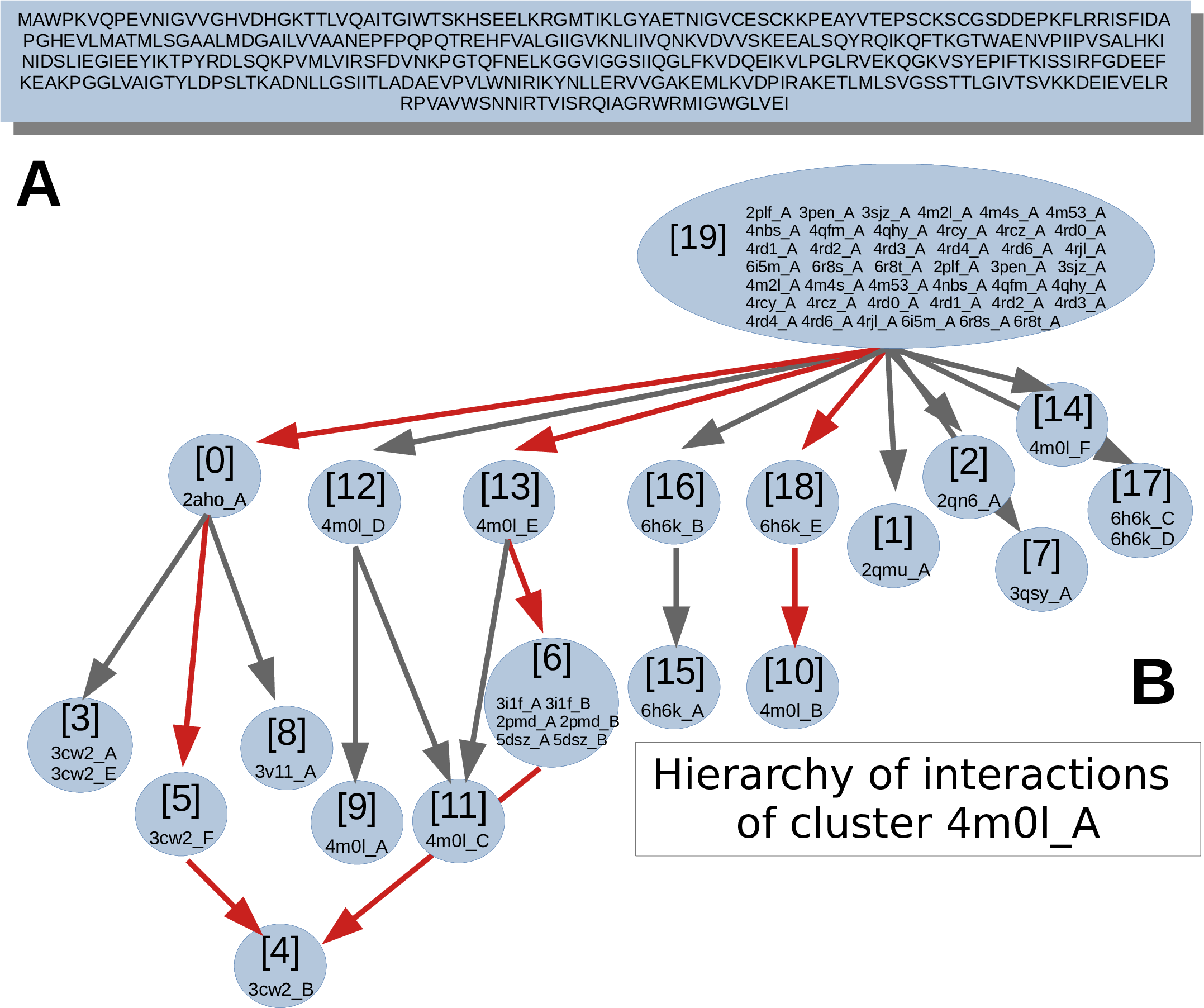

Supplement: S1 Fig — The blue circles group all chain structures contained in this node. Red edges highlight the “genealogical” relationships shown in Fig 1C. (TIF) [file pcbi.1010713.s001.tif]

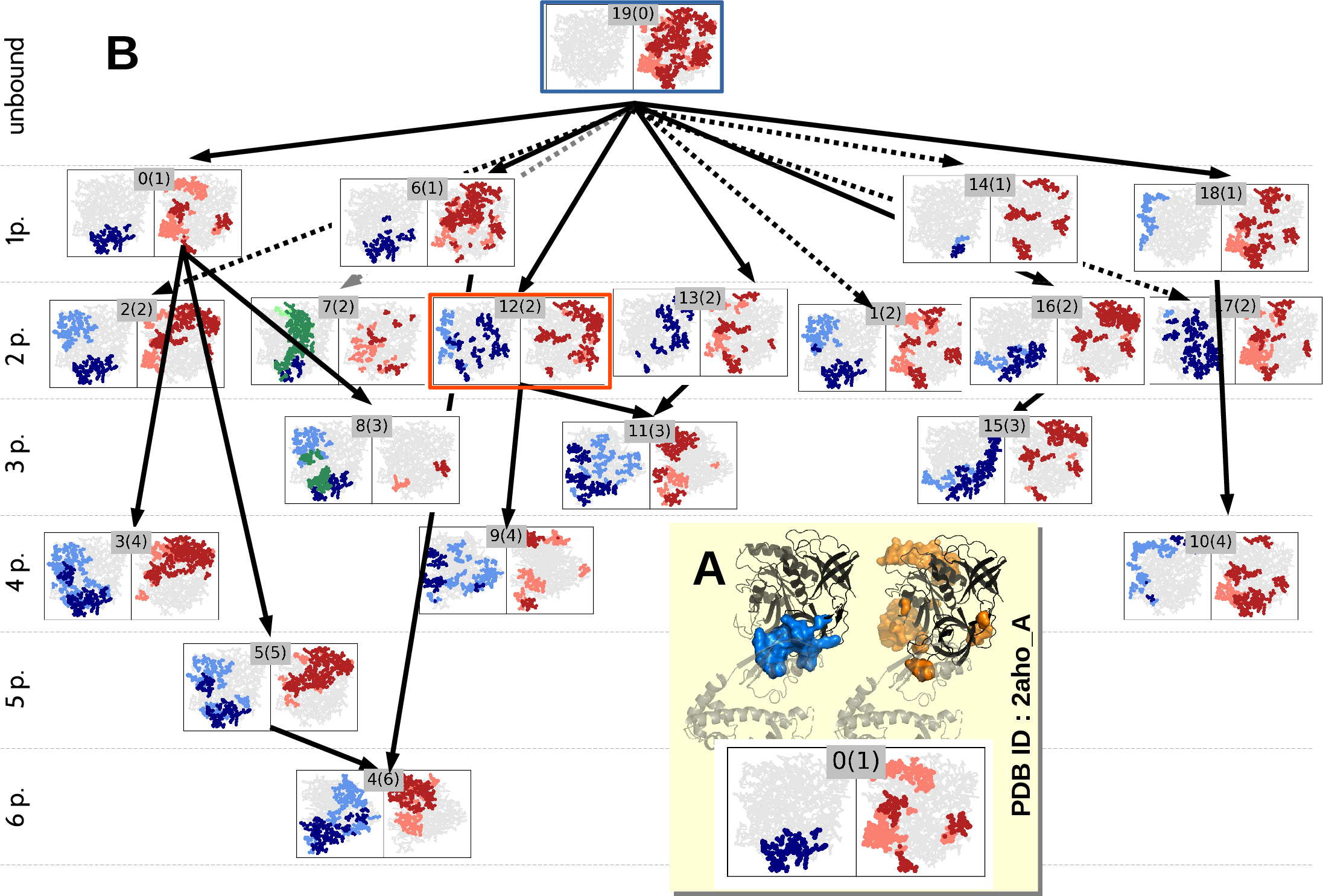

Supplement: S2 Fig — To improve visualisation, we have projected the 3-dimensional structures into a 2-dimensional sketch. Interface regions are shown in blue or green depending on whether the interaction is with another protein or with DNA, regions of soft disorder are coloured red, and residues that are forward are shown in a darker shade than those that are backward. Each node of the graph is labelled by two numbers “N(M)”, where N is the identifier of the node and M if the number of partners of the protein at node N. Note the input node of the graph, labelled 19(0), where M = 0 indicates the unbound form. A. An equivalence between the 3-dimensional representation of the protein and the sketch in 2-dimensions for the structure of node N = 0. In the 3D structures, the interfacial residues are shown in blue (top left) and the soft residues in orange (top right). B. The whole hierarchy of interactions from the PDB. Horizontal lines (Kp.) summarise all protein complexes with a fixed number of partners K. The arrows indicate the increase in the number of partners from top to bottom. With respect to the unbound structure in node 19(0) (blue square), all interactions contained in line 1p. define the 1-shell of interactions in Fig 1D. In the same way, all the interfaces in lines 1p. and 2p. define the 2-shell of interactions, and so on. The union of all interfaces in this graph forms the all-shell. If another predecessor node is considered as the origin, such as node 12(2) (orange square), its interaction shells would consist only of the nodes with a higher K connected to it. That is, node 11 for the 1-shell and nodes 9 and 11 for the 2-shell. (TIF) [file pcbi.1010713.s002.tif]

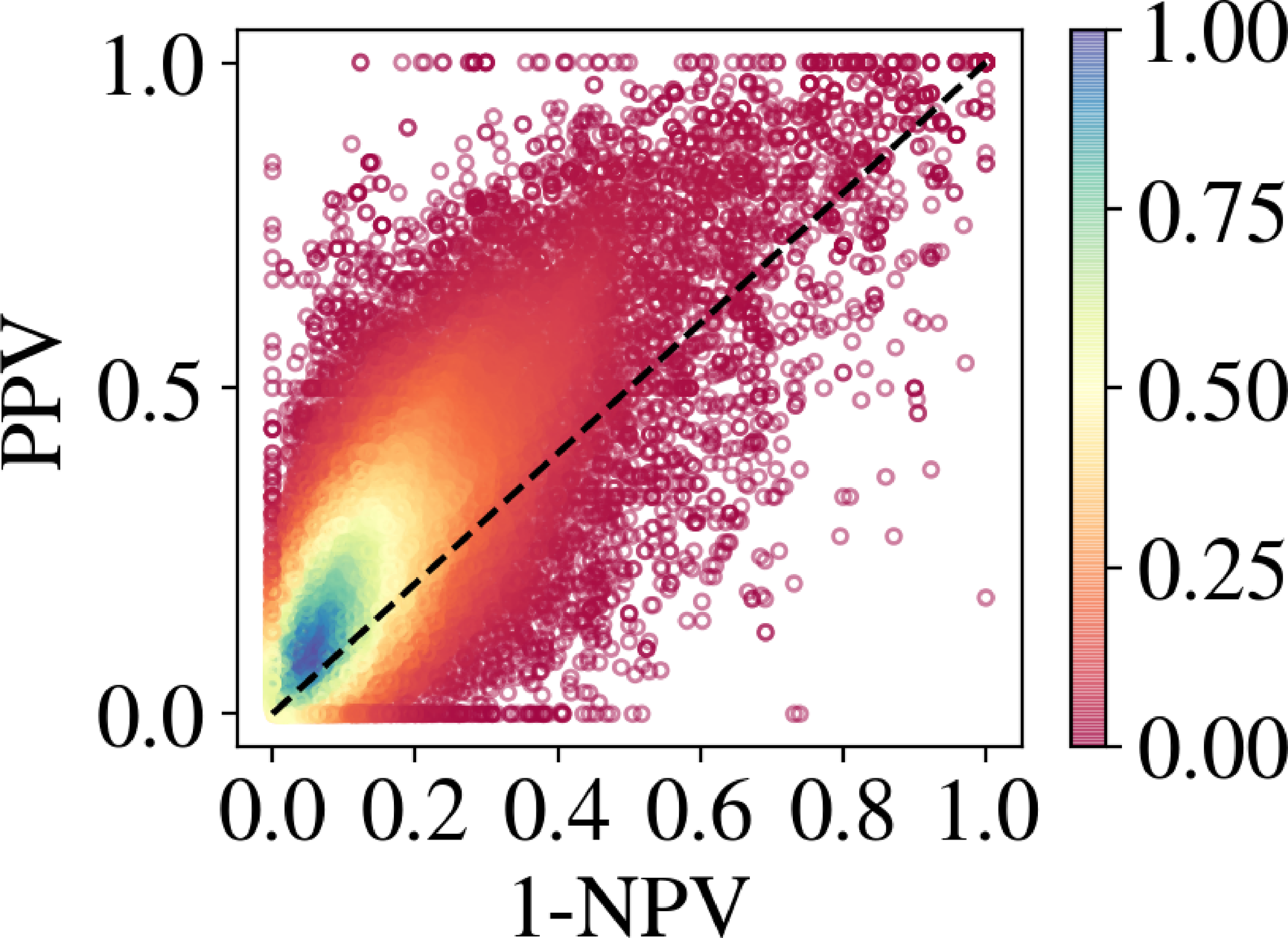

Supplement: S3 Fig — Each point corresponds to a prediction for a parent node. The colour encodes the local density of this region. As in Fig 2A, the 75% of the predictions lie above the random guess line. (TIF) [file pcbi.1010713.s003.tif]
