# Supplementary material for: Soft disorder modulates the assembly path of protein complexes
Source: PLoS Comput Biol. 2022 Nov 17;18(11):e1010713. doi: 10.1371/journal.pcbi.1010713 (PMC9714922; doi:10.1371/journal.pcbi.1010713)
Supplement: Attachment — Submitted filename: answer to referees.pdf [file pcbi.1010713.s004.pdf]

Dear Editor,

Enclosed please find the revision of our manuscript "Soft disorder modulates the assembly path of protein complexes", which we hereby submit for a second round of review. First, we would like to thank the reviewers for their careful and constructive reading of the previous version of the manuscript and for their positive feedback. Most of their concerns related to the clarity and conciseness of our previous presentation. Thanks to the reviewers' suggestions, we truly believe that our manuscript is now much easier to read and understand. We sincerely thank them for their help in improving the manuscript.

In what follows, we address all their comments and suggestions point by point. To summarize the changes:

- We improved the readability of the paper by changing it in its different parts and made the discussion shorter and less wordy. We also revised the English style. In particular, many details have been moved to the Methods and Materials section, and we have tried to keep the explanations short and simple. Some of the figures are now supplemental figures, and others have been combined at the suggestion of the reviewer.

- We have cited and discussed recent literature exploring the effects of flexibility on the assembly of protein complexes in both the introduction and the discussion.

- We explained why the term 'soft disorder' is used to cover contributions to binding by both flexibility and intermittent intrinsic disorder. Namely, 1. we clarified that we use both missing residues and high b-factor residues to define soft disorder, 2. we included an extra figure (Fig. 1 A) illustrating the definition, and 3. We explicitly discussed why both measures must be considered together for this study.

We hope you will like the current version of the article and look forward to your positive answer.

Best regards,

Beatriz Seoane   Alessandra Carbone

Reviewer's Responses to Questions

Comments to the Authors:

Please note here if the review is uploaded as an attachment.

Reviewer #1: The topic is interesting, and the conclusions potentially interesting. The only concern I have is that the IDR based interactions often happen with high on/off rates, and I am not sure to what extent they are involved in building up large complexes. The authors might want to comment on this in the discussion.

We thank the referee for considering the topic and the conclusions interesting. We agree with the referee that not all the IDRs are likely connected to protein interactions, and it was not explicitly stated or explained in the previous version of the manuscript.

In particular, we have added the following paragraph in the definition of soft disorder:

"*In this new work, we combine both kinds of disorder in the same soft disorder measure for the following reasons. In~\cite{seoane2021complexity} we have shown statistically that: (i)*

the majority of missing residues in the PDB were intermittently disordered/ordered residues when many crystals of the same protein were available, (ii) these disordered/ordered residues were also independently classified as high b-factor residues in protein clusters with more than 10 crystals in almost 100\% of cases, (iii) such residues were unlikely to be identified as missing by bioinformatic predictors of intrinsic disorder, even though hot loops are known to be easy to predict~\cite{linding2003protein}, and finally (iv) the missing residues excluded in our present analysis, the so-called ``always missing" regions in Ref.~\cite{seoane2021complexity} (i.e. regions that are missing in all structures of large clusters), were very accurately predicted by these predictors from the sequence. All these reasons justify considering missing residues that undergo disorder-to-order transitions and high b-factor residues, as two manifestations of the same effect, which leaves aside a group of intrinsic disordered residues whose role in the assembly is probably different and much harder to evaluate. Being these "always missing" residues easy to predict, they do not pose a problem to exclude them from the investigation of the role of soft disorder in protein assembly."

Furthermore, we added the following paragraph in the discussion section:

"*With regard to the role of intrinsic disorder, however, it is important to emphasise that our analysis formally considers only the missing residues that undergo a transition from disorder to order. In this sense, only two types of missing residues are included in the measure of soft disorder: the missing residues that are missing only in some of the protein structures of a node (structures with the same interaction complexity), e.g. hot loops, and residues that are missing in the parent structure but ordered in the progeny, i.e. regions that undergo a disorder-to-order transition. The residues that are missing in both the parent and the descendant structure are therefore ignored in our analysis, as there is no way to judge whether they belong to the interface of the progeny or not. It is unlikely that such regions are directly involved in the construction of large complexes, as they are never structured. In a previous work,~\cite{seoane2021complexity} we have shown that these ``always missing" regions are easily predicted from the sequence using predictors of intrinsic disorder, which means that they can be easily inferred and excluded for practical applications related to the assembly of complexes."*

In addition, the new version of the manuscript attempts to make our treatment more explicit in relation to intrinsic disorder including a new panel in Fig 1.

Reviewer #2: In this manuscript the authors study the role of protein flexibility in protein complex assembly. The work presented here follows from a previous study from the same authors (Seoane et al. PLOS Comp Bio 2021) where they have shown some degree of association between protein flexibility , estimated by a normalised B-factor, and interface residues. In the previous publication, these authors hinted at the possibility that flexibility could be predictive of interface regions along a path of assembly from monomeric to larger assemblies. This suggestion is now followed up statistically in the current manuscript.

Essentially, the single point of the manuscript is that interface residues of complexes having larger numbers of subunits can be somewhat predicted by the flexibility of residues in complexes having lower number of subunits. This result is shown in figure 5 with figures 6,7 and 8 essentially trying to show that the result is robust to different ways of looking at the

data. More broadly the relation between interface residues and flexibility has a very long history of study with quite many perspectives, including ideas around folding upon binding; flexibility of core vs. periphery of interface residues; and changes in entropy upon binding among other ideas and literature. The work done here, where the authors study changes in flexibility in a given protein with increasing numbers of bound interactors is a useful addition to the literature.

We would like to thank the reviewer for considering our work a "useful addition to the literature" and for all the precise suggestions.

I only have a small number of concerns and they are primarily related with presentation and clarity instead of technical issues:

1 - The biggest issue I have had while reviewing the paper was the clarity of the manuscript itself. There is essentially one main result in the paper but there are 9 figures with the fist real result starting in figure 5. Figures 1 to 4 are diagrams and a non critical result figure. Figures 6, 7 and 8 are controls and figure 9 is a very short result related with Alphafold predictions that probably should be removed. The text itself is also very difficult to follow because the authors introduce many acronyms and symbols (SDR, IR,k, K, PPV, etc) and some paragraphs are really full with acronyms and sentences one needs to re-read a few times to understand. It would be great if the authors could simplify and improve on the readability of the work.

We agree with the reviewer that the previous version of the manuscript was long and difficult to read. We have made great efforts to simplify the sentences, make it easier to discuss the results and improve the general style of the manuscript. We hope that the new version will be much easier for readers to follow. Furthermore, we have reduced the use of acronyms (especially when it comes to assessing the quality of the tests). We have also simplified the description of the tests in the hierarchy by introducing the language "parent-offspring" to avoid verbosity. We address the remaining comments immediately below.

1.1 – I suggest the authors could simplify and merge figures 1, 2 and 3 and move figure 4 to supplementary.

We did as the referee suggested. The new figure 1 contains old figures 1-3. In order to make some space, we moved old Fig.1B to the Supplementary File (now Fig S1). We also slightly modified Fig 1 to improve the comprehensibility of the analysis. Old Figure 4 is now Fig S2, as suggested.

1.2 – I don't really see the point of adding the Alphafold result at the end of the manuscript. It is a small example and as the authors state, it would require a proper analysis. I don't see how it adds to the central claim of the work. It would make more sense to develop into a proper stand alone piece of work. Instead, the authors could leave some of these ideas in the discussion section alone without any specific results.

We agree with the referee that the relationship between soft disorder and low confidence Alphafold regions needs to be carefully and statistically investigated in future work, but we do not think that the current presentation is uninteresting. In all the examples we have considered, the relative pLDDT is strongly correlated with soft disorder, particularly with the

union of soft disorder in the graph. This correlation suggests that the Alphafold predictor could in principle be used to exploit and better understand the effects of disorder in the assembly on a much broader scale, as Alphafold is not limited to known crystals. But not only that, these results also show the importance of correctly predicting the b-factor in intermediate protein complexes in order to correctly predict more complicated protein assemblies. In our opinion, deleting this section would severely limit future prospects and interest in this work.

1.3 – There are some parts of the results that could be moved to the methods section. Page 5 and 6 – the definitions of how the DAG was built could be, at least in part, moved to the methods. Page 9, around lines 202-206, the definition of random might be better in the methods.

We have followed the referee suggestions and we have moved the details of the graph construction, and on the correlation tests considered to the Methods and Materials section.

1.4 – It is hard to say what to do with the acronyms but, at least in places, the authors could try to avoid using them to make it easier for readers that are not as familiar with their terms.

We have tried to limit somewhat the use of acronyms, and in particular we avoid them in the captions to improve comprehensibility. We hope that the reviewer will find the current choice a good compromise between avoiding repetition and readability.

2 – One point that stood out from a technical perspective was that the authors have analysed the results in the form of how often the TPR and FPR are better than random. One of the key result is in Figure 5A where the authors report that 74% of the predictions (i.e. flexible regions predicting the next interface region) are better than random. While this seems to be the case I think it is also noteworthy that for the majority of these cases the predictions are really just somewhat better than random. For a given FPR most of the predictions have a TPR that is quite close but above random. Overall, this association reported in the manuscript seems real but having a small effect size. I think it would be important to comment on this in the discussion section.

We agree with the comment. We have explicitly mentioned this both in the presentation of the results and in the concluding discussion.

3 – The authors make a big point, both here and in the previous manuscript about the term "soft disorder". I don't really see the need to come up with a new term, why not just use the term flexibility ? Or relative flexibility since the authors used a protein normalized metric.

We use soft disorder because we do not count only flexible residues: Our definition includes both residues with a relatively high b-factor and missing residues that get structured either in alternative clusters of the same interaction complexity, or in offspring nodes. We need an umbrella term for both types of "disorder" and soft disorder seemed an intuitive choice. We have added several paragraphs in the new version of the manuscript (highlighted in red) to clarify our definition and the usefulness of using this measure. We have also added a new panel in Fig. 1 to clarify the definition of soft disorder. In addition, we must say that it is not true that high b-factor or missing regions are caused only by high flexibility. Rigid but amorphous structures also damage the quality of X-ray crystallography experiments.

Moreover, they would also provide a thermodynamic advantage to create short-lived interfaces, so they are very likely to be present in real proteins, as are the high-mobility regions. We have also endeavored to present parallel previous work highlighting the effects of intrinsic disorder and flexibility in protein complex assembly, thus justifying a joint consideration of the effects of both measures. These discussions can be found in the introduction, in the definition of soft disorder and in the conclusions.

Additional minor suggestions:

- It is not common to underline words in scientific communications, might be better to remove this

This was an unpleasant and unnoticed consequence of the PLOS format for the latex command “\em”. We have corrected this in the new version and the underlined sentences are now italicised.

- The abstract usually opens with some form of introductory sentence(s) as to why the work/topic is important.

Our abstract starts now with a long introductory sentence about the interest of this work.

Have the authors made all data and (if applicable) computational code underlying the findings in their manuscript fully available?

The PLOS Data policy requires authors to make all data and code underlying the findings described in their manuscript fully available without restriction, with rare exception (please refer to the Data Availability Statement in the manuscript PDF file). The data and code should be provided as part of the manuscript or its supporting information, or deposited to a public repository. For example, in addition to summary statistics, the data points behind means, medians and variance measures should be available. If there are restrictions on publicly sharing data or code —e.g. participant privacy or use of data from a third party—those must be specified.

Reviewer #1: None

Reviewer #2: Yes

PLOS authors have the option to publish the peer review history of their article (what does this mean?). If published, this will include your full peer review and any attached files.

If you choose “no”, your identity will remain anonymous but your review may still be made public.

Do you want your identity to be public for this peer review? *For information about this choice, including consent withdrawal, please see our Privacy Policy.*

Reviewer #1: No

Reviewer #2: No
