# Supplementary material for: Soft disorder modulates the assembly path of protein complexes
Source: PLoS Comput Biol. 2022 Nov 17;18(11):e1010713. doi: 10.1371/journal.pcbi.1010713 (PMC9714922; doi:10.1371/journal.pcbi.1010713)
Supplement: Attachment — Submitted filename: answer to referees - answer to referees.pdf [file pcbi.1010713.s005.pdf]

Dear Editor,

Enclosed please find the revision of our manuscript "Soft disorder modulates the assembly path of protein complexes". We have removed the duplicate paragraph as requested. We hope that this new version will be finally accepted for publication.

Best regards,

Beatriz Seoane   Alessandra Carbone
